# Bioinspired Synthesis of Graphene-Based Anatase TiO_2_ Nanoparticles/Nanorods Hierarchical Structure with Enhanced Capacity in Lithium-Ion Batteries

**DOI:** 10.3390/biomimetics10030144

**Published:** 2025-02-27

**Authors:** Zebang Yu, Hang Ping

**Affiliations:** State Key Laboratory of Advanced Technology for Materials Synthesis and Processing, Wuhan University of Technology, Wuhan 430070, China

**Keywords:** bioinspired synthesis, graphene, anatase, hierarchical structure, lithium-ion batteries

## Abstract

Titanium dioxide demonstrates promising potential in the energy storage field due to its high theoretical specific capacity and economic viability. However, its practical application is hindered by intrinsic limitations including low electronic conductivity and slow lithium-ion transport. In general, nature inspires the biotemplating synthesis of artificially functional materials with hierarchical structures. Learning from the bioinspired synthesis process, we adopt a facile biomimetic approach to synthesize graphene-based anatase TiO_2_ nanoparticle/nanorod hierarchical structure. The rod-shaped anatase is assembled nanoparticles with a diameter of 20 to 50 nm, and the surface of graphene is deposited by nanoparticles of 5 to 10 nm. The composite also possesses a high surface area and a mesoporous structure. This unique structure not only reduces the transportation pathway of lithium ions and electrons but also enhances the electric conductivity and tolerates the volume change. As an anode electrode, the bioinspired hierarchical structure exhibits a high reversible capacity of 160 mA h g^−1^ after 180 cycles at a current rate of 1C, highlighting the effectiveness of bioinspired design.

## 1. Introduction

Titanium dioxide (TiO_2_) has garnered significant scientific interest owing to its versatile physical and chemical properties, enabling applications spanning photocatalysis, coatings, dye-sensitized solar cells, lithium-ion batteries, and other fields [1,2,3,4]. As an emerging energy storage anode for Li-ion batteries, nanostructured titania demonstrates particular promise due to its high theoretical capacity, natural abundance, and cost-effectiveness [5,6]. Nevertheless, practical implementation faces critical challenges associated with sluggish lithium-ion diffusion kinetics and limited electronic conductivity during charge-discharge processes [7,8]. The sluggish movement of lithium-ion in TiO_2_ significantly impedes the efficiency of charge and discharge processes, resulting in longer charging times and reduced power output. Meanwhile, the low conductivity can result in poor electrical contact and inefficient electron transfer, further hampering the storage performance of the battery.

To address these limitations, strategic approaches involving conductive carbon integration and nanoscale engineering have been proposed, where carbon matrices enhance electron transport while nanostructure shortens ion diffusion pathways [9,10]. Carbonaceous materials, particularly graphene derivatives, have demonstrated exceptional potential for enhancing charge transfer kinetics due to their high intrinsic conductivity and large specific surface area [11]. Concurrently, nanostructuring effectively reduces Li^+^ diffusion lengths while increasing reactive sites. However, conventional synthesis routes for such hybrid architectures often involve energy-intensive processes or toxic reagents, raising sustainability concerns [12]. These challenges have stimulated growing interest in bioinspired synthesis approaches that mimic nature’s efficiency in creating hierarchical structures under benign conditions [13].

Compared with traditional chemical synthesis methods requiring harsh environments, biological systems, such as bacteria [14], virus [15] and biomolecules [16,17], offer unique advantages in templating nanoscale architectures through molecular recognition and biomineralization processes. Various research efforts have focused on using biomimetic methods to synthesize nanostructured titanium dioxide. For example, Hang et al. adopted genetically engineered *E. coli* as a three-dimensional framework to fabricate rod-shaped anatase assembled by nanoparticles with 5 nm diameter [18]. The organic matrices in bacteria also provide a carbon source to coat the surface of nanoparticles. While such biotemplating achieves precise nanostructural control, the inherent complexity of genetic engineering and limited scalability hinder practical implementation. Meanwhile, graphene-based composites have emerged as promising candidates due to their exceptional conductivity and large surface area which are useful for enhancing the lithium storage capacity [19,20,21]. However, achieving effective integration of inorganic components with graphene as anodes for lithium-ion batteries remains challenging. This limitation fundamentally originates from the inorganic components’ volume changes during charging and discharging cycles, which induce mechanical degradation and loss of electrical contact with graphene. To mitigate this, researchers have explored various strategies, including designing three-dimensional architectures, incorporating buffer layers, and using binder materials that accommodate volume changes without compromising the overall structure.

**Figure 1:**
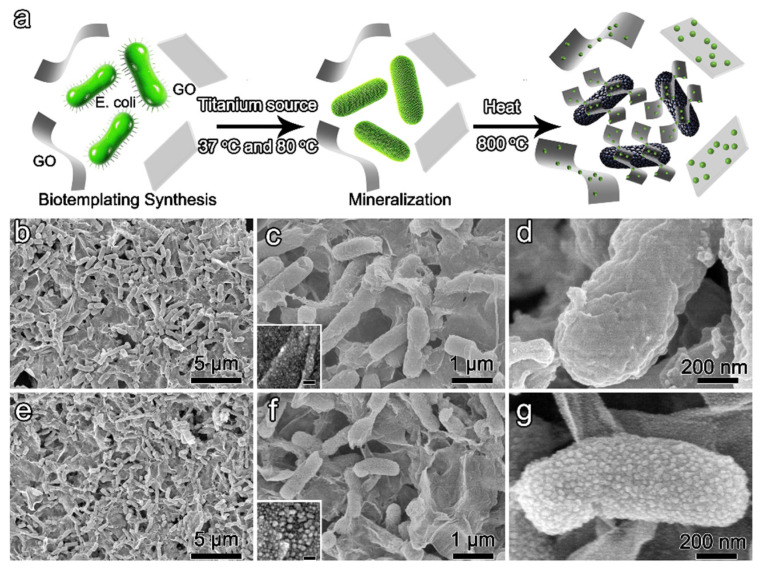
(**a**) Bioinspired biotemplating synthesis; (**b**–**d**) SEM images of TiO_2_@C under successive incubation at 37 °C and 80 °C; (**e**–**g**) SEM images of above products after heat treatment at 800 °C in Ar. Scale bar in inset is 50 nm.

In this work, we propose a facile bioinspired approach to synthesize graphene-based anatase TiO_2_ nanoparticles/nanorods hierarchical structure (Figure 1a). Initially, Graphene oxide was dispersed in water to combine with *E. coli*, and uniformly wrapped around bacteria under the driving force of electrostatic interactions. Meanwhile, titanium source deposited on the surface of bacteria and transformed into titania precursor at low temperature. After heat treatment in an inert atmosphere, graphene oxide was transformed into graphene and the mineralized bacteria transformed into rod-shaped anatase assembled by nanoparticles. Notably, nanoparticles with an average 7 nm diameter can be deposited on graphene through chemical vapor deposition of a precursor. The hierarchical structure exhibits synergistic advantages: graphene’s conductive network enables rapid electron transport, the nanorod morphology ensures structural stability, and surface nanoparticles provide abundant active sites while maintaining short ion diffusion paths. This bio-templating approach presents a sustainable pathway for designing advanced electrode architectures, combining nature-inspired self-assembly with nanoscale engineering to address common challenges in energy storage materials, including poor conductivity and volume expansion issues.

## 2. Materials and Experiments

### 2.1. Synthesis of Nanostructured Anatase

The wild-type *Escherichia coli* (BL21(DE3)) was initially cultured in Luria–Bertani (LB) medium supplemented with 30 μg mL^−1^ kanamycin under constant agitation (37 °C, overnight). The cell suspension was diluted in fresh LB medium and incubated at 37 °C for 4 h with shaking. Cells were collected via centrifugation at 8000× *g* and 4 °C, resuspended in 10 mL TBS buffer (50 mM Tris–HCl, pH 7.0, 300 mM NaCl, Sigma-Aldrich, St. Louis, MO, USA). A titanium (IV) bis (ammonium lactato) dihydroxide (TiBALDH, Sigma-Aldrich, St. Louis, MO, USA) solution (2 mL) was slowly added to the suspension. The mixture underwent mineralization via sequential incubation at 37 °C (24 h, gentle shaking) and was transferred into a water bath at 80 °C (24 h, static conditions). The mineralized products were isolated by centrifugation, rinsed with deionized water, and lyophilized. Nanostructured anatase was obtained by annealing in a tubular furnace at setting temperatures (700 °C, 800 °C, 900 °C or 1000 °C) for 4 h with a heating rate of 4 °C min^−1^.

### 2.2. Preparation of TiO_2_@Graphene Composites (TiO_2_@C)

Graphene oxide solution with a concentration of 1 mg mL^−1^ was synthesized via a modified Hummers method [22]. *E. coli* cell pellets were dispersed in 10 mL TBS buffer (50 mM Tris-HCl, pH 7.0, 300 mM NaCl), followed by sequential addition of 1 mL graphene oxide solution and 2 mL titanium (IV) bis(ammonium lactato) dihydroxide solution (TiBALDH, Sigma-Aldrich, USA). The mineralization protocol followed the aforementioned procedure. Mineralized composites were isolated by centrifugation and washed with deionized water, then dried in a lyophilizer. Graphene-based anatase TiO_2_ nanoparticle/nanorod hierarchical structure was synthesized by annealing in a tubular furnace at 800 °C for 4 h with a heating rate of 4 °C min^−1^.

### 2.3. Characterization of Nanostructured Anatase

X-ray diffraction (XRD) patterns were conducted on Bruker D8 Advance with Cu Kα radiation (V = 40 kV, I = 40 mA) in the range of 20–80° (Empyrean, Panalytical, The Netherlands). Surface morphology information was revealed by field emission scanning electron microscopy (FESEM) in a Hitachi S-4800 at 5 kV equipped with an energy-dispersive spectroscopy (EDS) detector (Amsterdam, The Netherlands). High-resolution transmission electron microscopy (HRTEM) images were acquired using a JEOL JEM 2100F microscope (Akishima-shi, Tokyo, Japan) at 200 kV. The specific surface area was calculated via an ASAP 2020M adsorption apparatus (Norcross, GA, USA) using the Brunauer–Emmet–Teller (BET) method. The Raman spectrum was acquired using a Renishaw InVia Raman spectrometer (Gloucestershire, London, United Kingdom) equipped with a 785 nm laser for the measurements. Thermogravimetric (TG) profiles were obtained using a Netzsch STA449F3 analyzer (Selb, Germany) at a heating rate of 10 °C min^−1^ from 40 °C to 900 °C. X-ray photoelectron spectroscopy (XPS) data were collected on a ThermoFisher Escalab 250Xi (Waltham, MA, USA).

### 2.4. Electrochemical Measurements

CR2025-type coin cells were constructed with lithium metal foil as the counter and reference electrode for electrochemical testing. To fabricate the working electrode, a N-methyl-2-pyrrolidone (NMP, Aladdin, Shanghai, China) slurry of active materials was mixed with Super P carbon black and polyvinylidene fluoride in a weight ratio of 7:2:1. After thorough grinding, the resultant slurry was uniformly coated onto Cu foil and vacuum dried at 120 °C for 12 h. The mass loading of active materials in each coin cell was approximately 1.5 mg. Coin cells were assembled in an Ar-filled glove box, employing 1 M lithium hexafluoro-phosphate in ethylene carbonate (EC)/diethyl carbonate (DEC) (1:1 *v*/*v*) as the electrolyte solution. Celgard polypropylene was used as the separator. The charge-discharge experiments were performed using a LAND battery tester CT2001A with a voltage window of 1–3 V (vs. Li^+^/Li) at multiple current densities (1C = 167 mA g^−1^). The electrochemical impedance spectroscopy (EIS) analysis was conducted using Autolab PGSTAT 302N equipment across the frequency range from 100 kHz to 0.01 Hz. A cyclic voltammetric (CV) test was performed in an electrochemical workstation in the range of 1–3 V (vs. Li^+^/Li) at varying scanning rates.

## 3. Results and Discussion

**Figure 2:**
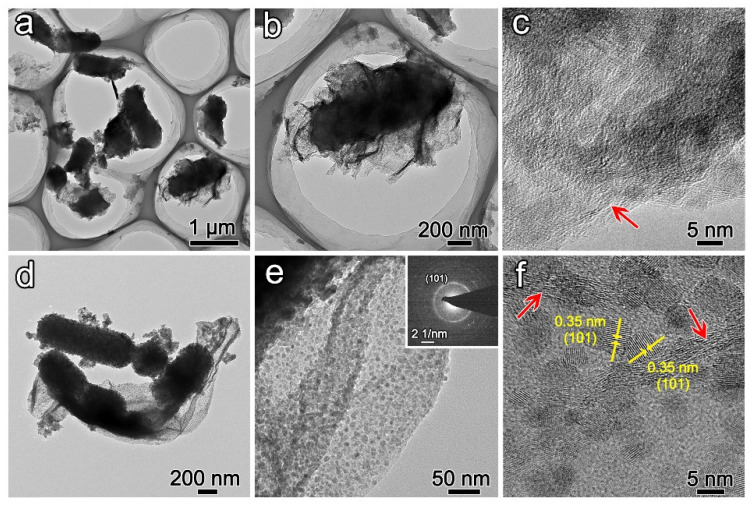
(**a**–**c**) TEM images of TiO_2_@C under successive incubation at 37 °C and 80 °C; (**d**–**f**) TEM images of above products after heat treatment at 800 °C in Ar.

The synthesized graphene oxide (GO) exhibits strong hydrophilicity and forms a stable aqueous solution at room temperature. The negatively charged GO surface enables homogeneous mixing with rod-shaped *Escherichia coli* (*E. coli*) through electrostatic repulsion equilibrium between two negatively charged systems (bacterial membrane and GO). Through interacting *E. coli*/GO with titanium source at 37 °C and 80 °C successively, the mineralized bacteria are uniformly distributed around GO (Figure 1b). TEM images demonstrate that bacteria are wrapped by GO (Figure 2a,b). The edge of GO is marked by a red arrow in Figure 2c. The smooth surface of GO is similar to the original GO (Figure 1c inset and Figure S1). There is no deposition of particles on the surface of GO, indicating no interaction between the titanium source and GO. The retained rod-shaped structure of bacteria indicates that the titania precursor deposits on its surface (Figure 1c). Otherwise, the structure of bacteria would be collapsed after freeze-drying. The rough surface on bacteria also verifies the deposition of precursor (Figure 1d). In the outer membrane of bacteria, some functional groups in the phospholipid bilayer may promote the hydrolysis and condensation of titanium source [23,24]. The precursor may first deposit on the surface of bacteria and then break the cell membrane to enter into the intracellular compartment of bacteria. Therefore, the rod-shaped structure can be maintained.

After heat treatment of previous products at 800 °C in Ar for 4 h, the graphene-based hierarchical structure is obtained. Due to the decomposition of organic matrices in bacteria, the diameter of the rod structure is decreased (Figure 1e,f). The GO would transform to graphene with a smooth surface through the removal of the hydrophilic group. However, the surface of graphene is anchored by numerous nanoparticles with diameters ranging from 5 nm to 10 nm (Figure 1f inset). The nanoparticles may result from the chemical vapor deposition of the precursor. Meanwhile, the rod-shaped structure is assembled by nanoparticles with diameters of 20 nm to 50 nm (Figure 1g). TEM image demonstrates the distribution of nanoparticles on the surface of graphene, SAED pattern confirms the polycrystalline nature of nanoparticles (Figure 2d,e). The measured d-spacing of 0.35 nm corresponds to the (101) crystallographic plane in anatase (Figure 2f). Direct mixing of pre-formed anatase rods with GO produces inhomogeneous composites (Figure S2). Because the surface of anatase is more rigid than native bacteria, it is hard to combine with GO.

**Figure 3:**
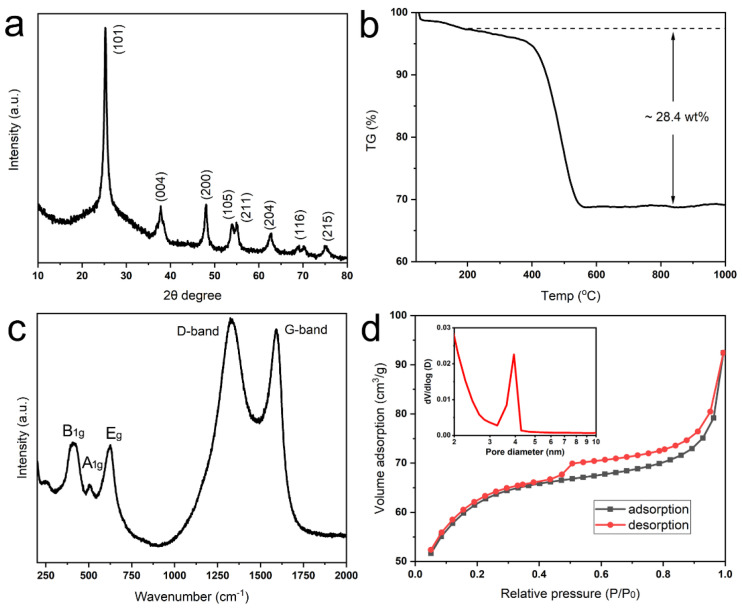
Composition of TiO_2_@C after annealing at 800 °C, 4 h. (**a**) XRD pattern, (**b**) Thermo gravimetric analysis; (**c**) Raman spectrum; (**d**) Nitrogen adsorption and desorption isotherms and pore size distribution (inset).

The crystallographic phase of annealed products with strong peaks is indexed to the (101), (004), (200), (105), (211), and (204) crystal planes of anatase TiO_2_ (JCPDS no. 21-1271) by XRD analysis (Figure 3a). Whereas there is only a broad peak around 25°, indicating the amorphous nature of products without heat treatment (Figure S3). The carbon content of TiO_2_@C is about 28.4 wt% (Figure 3b), and in pure carbon-coated rod-shaped anatase, it is 13 wt%. Therefore, the content of graphene is about 15 wt% in composite. The Raman spectrum shows characteristic D-band and G-band peaks at 1325 cm^−1^ (disorder carbon) and 1590 cm^−1^ (graphitic carbon), respectively (Figure 3c) [25]. Three additional peaks at 390 cm^−1^ (B_1g_), 508 cm^−1^ (A_1g_) and 630 cm^−1^ (B_1g_) are characteristic of the anatase phase [25]. The nitrogen adsorption-desorption isotherm (IV type) indicated the mesoporous structure with the main pore size around 4 nm, which is derived from the Barrett–Joyner–Halenda (BJH) method (Figure 3d inset). The pore volume and specific surface area are determined as 0.14 cm^3^ g^−1^ and 222.8 m^2^ g^−1^ (Figure 3d). Through annealing the pure mineralized rod-shaped products, it is also assembled by anatase TiO_2_ nanoparticles (Figure S4), and the pore volume and specific surface area are 0.136 cm^3^ g^−1^ and 69.9 m^2^ g^−1^ (Figure S5). The high specific surface area is ascribed to the combination of graphene and nanosized particles, and pore volume is resourced from the decomposition of organic matter.

**Figure 4:**
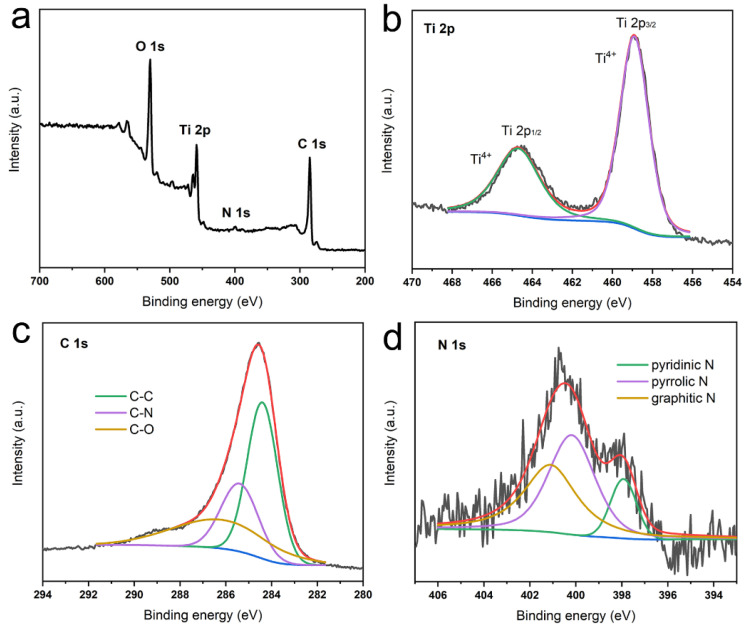
XPS spectra of TiO_2_@C after annealing at 800 °C, 4 h. (**a**) Full spectrum, high-resolution XPS spectra of (**b**) Ti 2p, (**c**) C 1s and (**d**) N 1s.

The XPS spectrum of TiO_2_@C is presented in Figure 4a, the peaks are ascribed to O 1s, Ti 2p, N 1s, and C 1s. The high-resolution of Ti 2p contains two peaks located at 458.9 and 464.7 eV are assigned to Ti 2p_3/2_ and Ti 2p_1/2_, respectively (Figure 4b) [26]. The fitted peaks in the high resolution of C 1s spectrum at binding energies of 284.2, 285.4, and 286.2 eV are attributed to C–C, C–N, and C–O bonds, respectively (Figure 4c) [12]. From the high-resolution spectrum of N 1s, the distinct peaks are ascribed to pyridinic N (397.9 eV), pyrrolic N (400.2 eV), and graphitic N (401.1 eV) (Figure 4d). Of the three nitrogen configurations discussed, only pyrrolic nitrogen has been reported to enhance the electrical conductivity of graphene films. Pyridinic nitrogen, characterized by a localized electron pair, is invariably associated with vacancy defects. Graphitic nitrogen typically exists at nitrogen-containing dangling bonds or as a physiosorbed species, neither of which can restore the hexagonal graphitic structure [27]. The nitrogen element doping from bacterial biomolecules enhances carbon’s electrical conductivity. Through annealing the mineralized products under different temperatures, the structure and phase are presented in Figures S6 and S7. There is no deposition of nanoparticles on the surface of graphene at 700 °C, it is not enough to generate the chemical vapor deposition of precursor. At 900 °C and 1000 °C, the mixed phases of anatase and rutile coexist in products, and the larger particles are around graphene.

**Figure 5:**
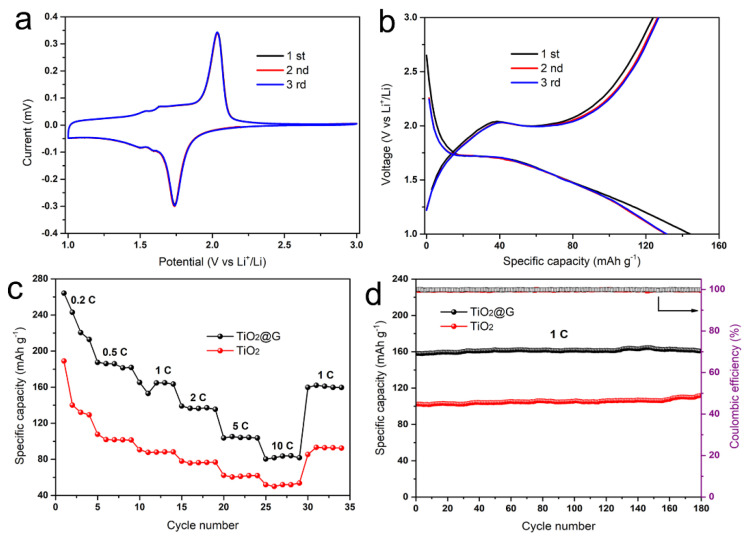
Electrochemical performance of graphene-based anatase electrodes. (**a**) Cyclic voltammetry curves with a scan rate of 0.2 mV s^−1^; (**b**) Voltage-capacity curves of the above electrode at a current rate of 0.2 C; (**c**) Rate capability of electrodes at various current rates; (**d**) Cycling performance of electrodes at a current rate of 1C.

Owing to the hierarchical TiO_2_@C architecture, its electrochemical performance was assessed through application as a lithium-ion battery anode. The initial three cycles of cyclic voltammetry (CV) curves of anode at a scanning rate of 0.2 mV s^−1^ are presented in Figure 5a. In the voltage range of 1.0 V to 3.0 V, there are two strong peaks located at about 1.75 V and 2.0 V. These processes correspond to Li^+^ intercalation/deintercalation within the anatase lattice. The almost overlapping of cycling CV curves means the high stability of this composite. The cycling voltage behavior of the electrode at a current rate of 2C is presented in Figure 5b. In the first cycle, the specific discharge and charge capacities are 144 and 125 mA h g^−1^. The 13% irreversible capacity loss is ascribed mainly to the formation of a solid electrolyte interface (SEI) layer, such as decomposing liquid electrolyte or trapping lithium ions [28]. In the following second and third cycles, the discharge and charge curves exhibit the same behavior, indicating the good stability of the anode again. The discharge profile of voltage changes can be divided into three regions: (I) from open circuit potential to 1.75 V, the sharp voltage decline corresponds to homogeneous Li^+^ intercalation within the anatase lattice; (II) the plateau at around 1.75 V presents the coexistence of Li-rich and Li-poor phases; (III) the gradual voltage decline beyond 1.75 V signifies Li^+^ accumulation at surface/interfacial sites of anatase TiO_2_ nanoparticles [29].

The rate capability of TiO_2_@C and pure anatase (TiO_2_) is investigated by various current rates from 0.2C to 10C (Figure 5c). The discharge capacities of TiO_2_@C are 242, 184, 165, 142, 103, and 82 mA h g^−1^ at each rate, which are higher than those of TiO_2_ anode (130, 102, 87, 78, 64, and 47 mA h g^−1^). After being cycled at a high rate of 10C, the TiO_2_@C anode could recover to 166 mA h g^−1^ at 1C. The electrodes’ cycling performance was evaluated under a 1C current rate. Both of them exhibit good capacity retention after 180 cycles (160 mA h g^−1^ for TiO_2_@C, 103 mA h g^−1^ for TiO_2_). The discharge capacities of graphene-based anatase electrodes without heat treatment show the lowest rate capabilities (65, 33, 24, 19, 11, and 7 mA h g^−1^) and cycling ability (24 mA h g^−1^ after 180 cycles at 1C) (Figure S8). However, there was a rapid capacity drop during the first four cycles at 0.2C. There are several potential factors contributing to the capacity drop observed during the initial cycles. (i) Formation of the solid electrolyte interface (SEI) film. During the initial charge and discharge process, the electrolyte undergoes reduction reactions on the anode surface (such as graphite), leading to the formation of the SEI film. This process consumes active lithium ions, resulting in an irreversible capacity loss during the first cycle, typically ranging from 5% to 20%. In the following cycles, the SEI film may not be fully stabilized, and in certain regions, side reactions (such as electrolyte decomposition) continue to occur. These reactions further consume lithium ions or electrolyte components, leading to a gradual decline in capacity. (ii) Electrolyte consumption and side reactions. At low charging rates (0.2C), the battery may experience prolonged charge-discharge cycles, which can intensify side reactions between the electrolyte and electrodes (such as oxidation or reduction decomposition). These reactions generate gases or inert products and consume active materials, leading to a decline in overall performance. (iii) Deterioration of interface contact. Repeated charge-discharge cycles can lead to a reduction in the adhesion between the electrode material and the current collector (such as copper foil). This degradation increases the interface resistance, thereby reducing the effective capacity of the battery.

Furthermore, the increase in capacity of the TiO_2_ anodes after 160 cycles was observed. It could be attributed to several interrelated mechanisms that enhance lithium-ion storage and electrochemical performance over prolonged cycling. (i) Structural activation. TiO_2_ may undergo phase transformations (e.g., from anatase to a lithium titanate phase) during cycling, creating a structure with better ion diffusion pathways or higher theoretical capacity. Repeated lithiation/delithiation can induce partial amorphization, increasing the number of active sites for lithium insertion. (ii) Particle size reduction. Mechanical stress from cycling fractures TiO_2_ particles into smaller nanoparticles, increasing surface area and shortening lithium-ion diffusion paths. This enhances reaction kinetics and accessible capacity. (iii) Solid-electrolyte interphase (SEI) optimization. Initial SEI layers may be resistive, but prolonged cycling stabilizes the SEI into a more ion-conductive and mechanically robust form, reducing impedance over time. (iv) Electrode morphology and contact improvements. Binder redistribution or conductive additive rearrangement improves electrical contact between particles, enhancing overall electrode conductivity. Particle cracking may expose fresh surfaces, improving electrolyte penetration and active material utilization.

The bioinspired hierarchical structure of TiO_2_@C contributes to its higher lithium storage capability and improved stability. First, the nanoscale architecture comprising graphene-supported nanoparticles and anatase nanorods shortens Li^+^ ion diffusion pathways within the crystalline matrix, significantly enhancing rate capability and cycling stability relative to bulk electrodes. Meanwhile, the nanoparticle-assembled nanorod architecture prohibits their stacking during lithiation and delithiation. The graphene-based anatase hierarchical architecture demonstrates significantly enhanced specific surface area compared to conventional nanorod configurations. The hierarchical structure could reduce aggregation and maximize exposed active sites, to further enhance ion transport. Because the hierarchical porosity shortens Li^+^ diffusion distances and enables rapid electrolyte penetration into the electrode bulk. Conventional TiO_2_ lacks such interconnected porosity, leading to underutilized regions (“dead zones”) and sluggish kinetics.

Second, the existence of graphene will not only enhance the intrinsic conductivity of anatase and suppress the formation of SEI but also provide a high surface area for contact with liquid electrolytes. Benefiting from graphene’s high electrical conductivity, the nanoparticles deposited on graphene enhance electron transport kinetics and reduce charge transfer resistance, thereby improving overall electrochemical performance. The combined effect of nanoparticle size reduction and graphene’s two-dimensional architecture provides an elevated surface area, offering abundant active sites for lithium-ion storage. In contrast, nanoparticle-assembled nanorods exhibit relatively lower specific surface area, which may restrict their rate capability. Third, mesoporous architecture effectively accommodates volumetric strain during Li^+^ intercalation/deintercalation while enhancing ionic mobility through interconnected channels and preserves electrode integrity. Therefore, a graphene-based hierarchical structure is beneficial for high capacity and enhanced stability. By combining “high specific surface area” and “optimized ion transport pathways”, their synergy addresses key limitations of conventional materials, such as sluggish kinetics and poor electrolyte accessibility, making hierarchical architectures ideal for high-power applications like fast-charging batteries. The design principles of hierarchical structures-balancing porosity, conductivity, and mechanical stability-are critical for advancing next-generation energy storage systems.

While the bio-templated synthesis of TiO_2_@C hierarchical structures demonstrates promising electrochemical performance, its large-scale implementation still faces multifaceted challenges: (i) Bacteria capable of surface mineralization are cultivated under controlled conditions to ensure they have the necessary properties for subsequent steps. Selecting and maintaining these bacteria requires specialized knowledge and equipment, as different strains vary in efficiency. (ii) The cultivated bacteria are then incubated with titanium sources. This step is critical because titanium compounds form the desired material structure. Factors like pH, temperature, and titanium concentration influence this interaction, so optimal conditions are essential for uniform mineralization. (iii) The mineralized bacteria are combined with graphene. Graphene enhances the material’s electrical conductivity and mechanical strength, but precise techniques are needed to ensure proper dispersion and bonding. Inconsistencies can compromise the material’s quality and functionality. (iv) The composite undergoes high-temperature treatment to stabilize its structure and improve electrochemical properties. Temperature and duration must be carefully controlled to avoid damage or altering characteristics. This process also removes residual organic components from the bacteria, resulting in a robust and efficient material suitable for lithium-ion batteries.

## 4. Conclusions

In summary, we utilized a bioinspired templating approach to synthesize a graphene-based hierarchical structure composed of anatase TiO_2_ nanoparticles and nanorods with enhanced lithium storage performance. After annealing, the rod-shaped anatase was uniformly encapsulated by graphene. The nanoparticles were deposited on the graphene surface via chemical vapor deposition of titanium dioxide precursors. As an anode electrode material, this composite integrates nanoparticles, mesoporous structures, and graphene, effectively addressing the intrinsic issues of poor electronic conductivity and lithium-ion transport. The electrode exhibits a high reversible capacity of 160 mA h g^−1^ after 180 cycles at a current rate of 1C. This bioinspired strategy may broaden the scope and impact of nanostructured materials in energy storage applications.

## Data Availability

The original contributions presented in this study are included in the article. Further inquiries can be directed to the corresponding authors.

## Supplementary Materials

The following supporting information can be downloaded at: https://www.mdpi.com/article/10.3390/biomimetics10030144/s1, Figure S1: SEM images of (a,b) graphene oxide and (c,d) graphene; Figure S2: Mixing with anatase and graphene oxide directly. (a) Optical image of products after freeze drying; (b–d) SEM images of distribution of rod-shaped anatase and graphene oxide; Figure S3: XRD pattern of TiO_2_@C under successive incubation at 37 °C and 80 °C; Figure S4: TEM images of rod-shaped TiO_2_ after annealing at 800 °C, 4 h; Figure S5: Nitrogen adsorption and desorption isotherms and pore size distribution (inset) of rod-shaped TiO_2_ after annealing at 800 °C, 4 h; Figure S6: SEM images of TiO_2_/TiO_2_@C under different heat treatment. (a,b) 700 °C, 4 h, (c,d) 900 °C, 4 h, (e,f) 1000 °C, 4 h; Figure S7: XRD patterns of TiO_2_/TiO_2_@C after annealing at various temperatures; Figure S8: Electrochemical performance of graphene-based anatase electrode under successive incubation at 37 °C and 80 °C. (a) Rate capability of electrodes at various current rates. (b) Cycling performance of electrodes at a current rate of 1C.
