# Supplementary material for: Bioinspired Synthesis of Graphene-Based Anatase TiO2 Nanoparticles/Nanorods Hierarchical Structure with Enhanced Capacity in Lithium-Ion Batteries
Source: Biomimetics (Basel). 2025 Feb 27;10(3):144. doi: 10.3390/biomimetics10030144 (PMC11940773; doi:10.3390/biomimetics10030144)
Supplement: Supplementary file 1 [file biomimetics-10-00144-s001.zip › biomimetics-3496439-supplementary.pdf]

Supporting Information

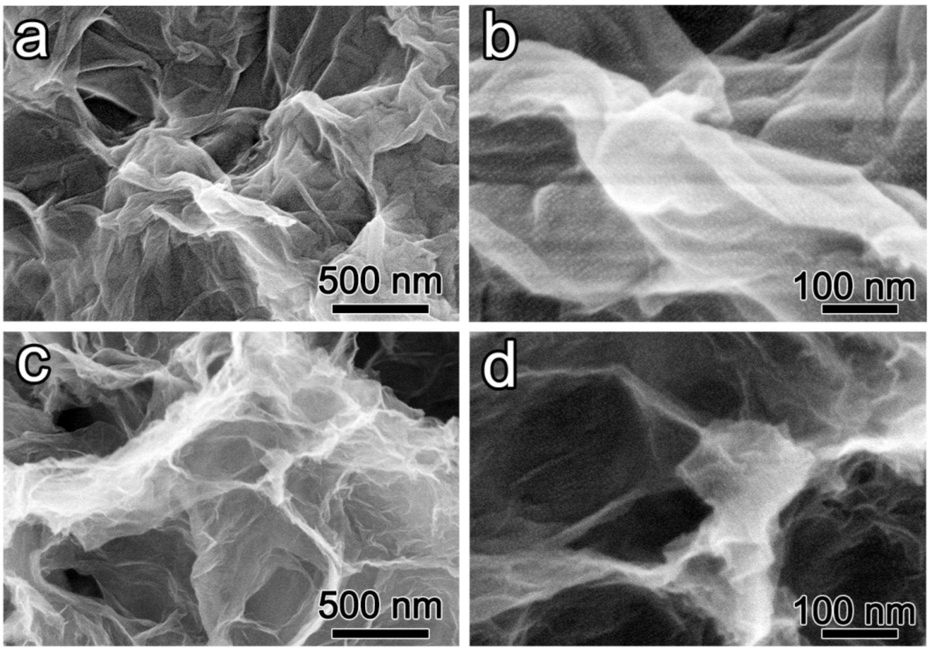

Figure S1. SEM images of (a-b) graphene oxide and (c-d) graphene.

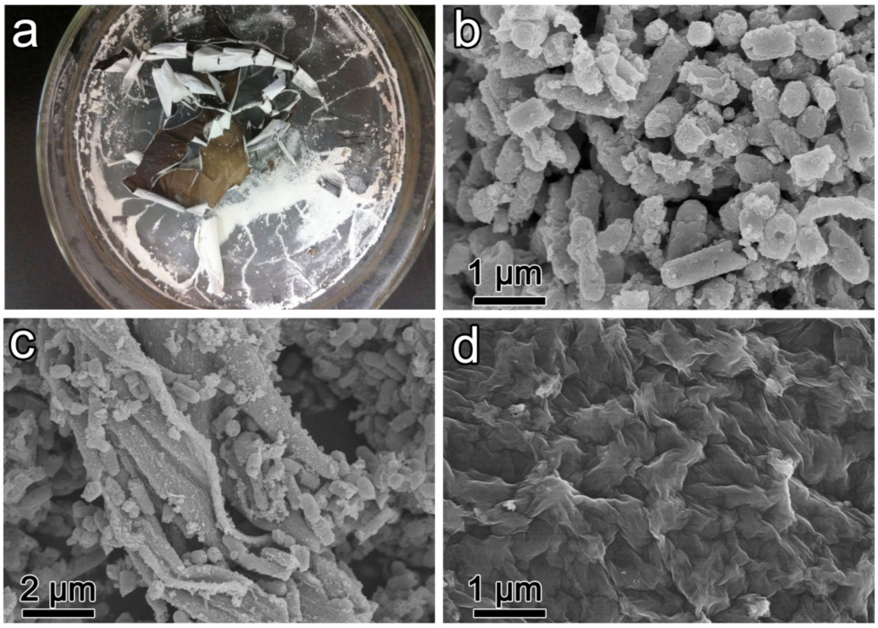

Figure S2. Mixing with anatase and graphene oxide directly. (a) Optical image of products after freeze drying, (b-d) SEM images of distribution of rod-shaped anatase and graphene oxide.

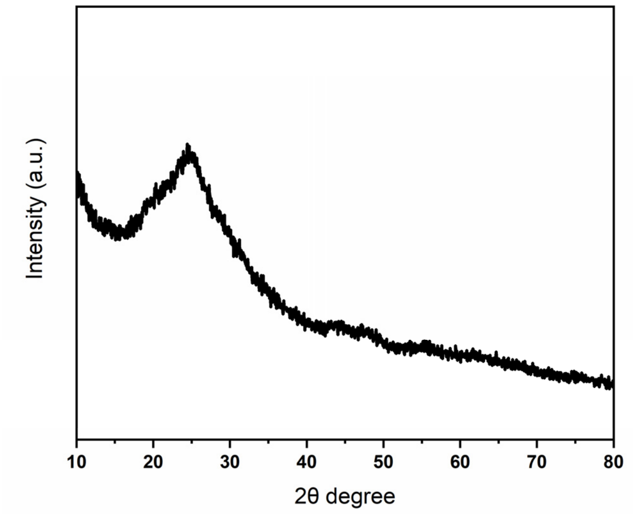

Figure S3. XRD pattern of TiO_2@C under successive incubation at 37 °C and 80 °C.

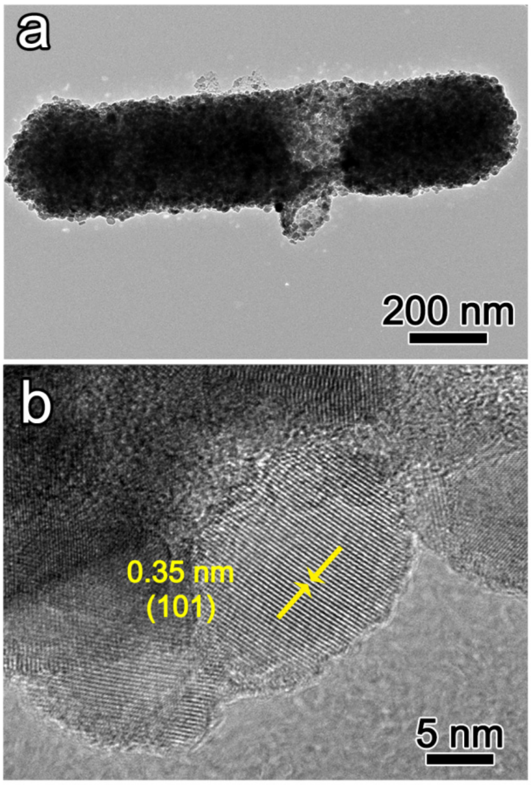

Figure S4. TEM images of rod-shaped TiO_2 after annealing at 800 °C, 4 h.

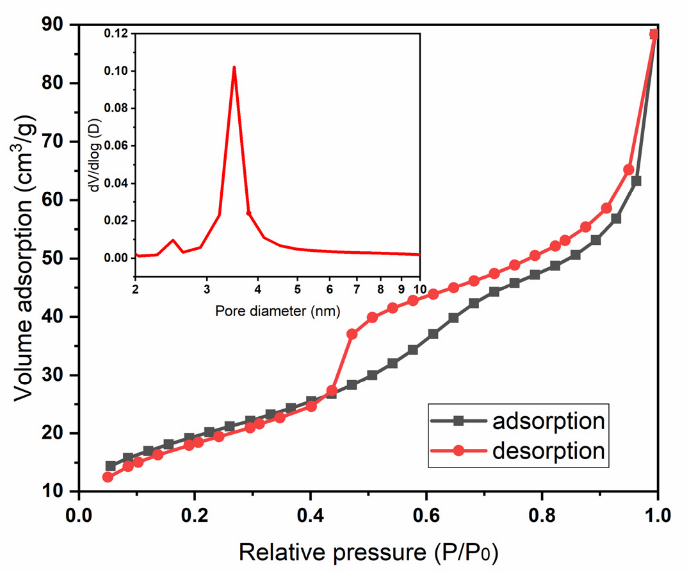

Figure S5. Nitrogen adsorption and desorption isotherms and pore-size distribution (inset) of rod-shaped TiO_2 after annealing at 800 °C, 4 h.

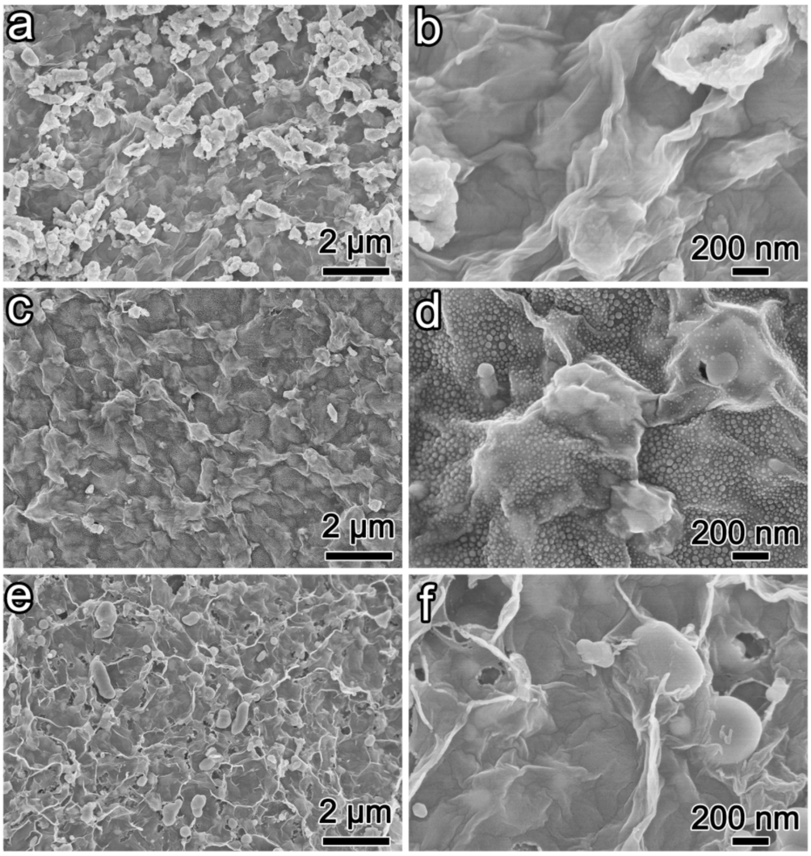

Figure S6. SEM images of TiO_2/TiO_2@C under different heat treatment. (a-b) 700 °C, 4h, (c-d) 900 °C, 4h, (e-f) 1000 °C, 4h.

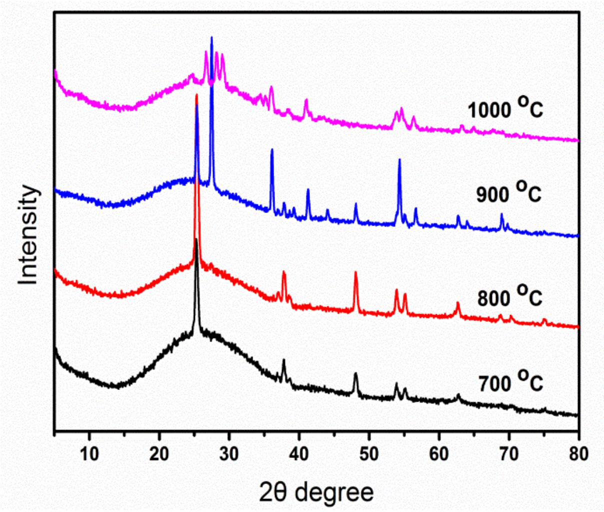

Figure S7. XRD patterns of TiO_2/TiO_2@C after annealing at various temperature.

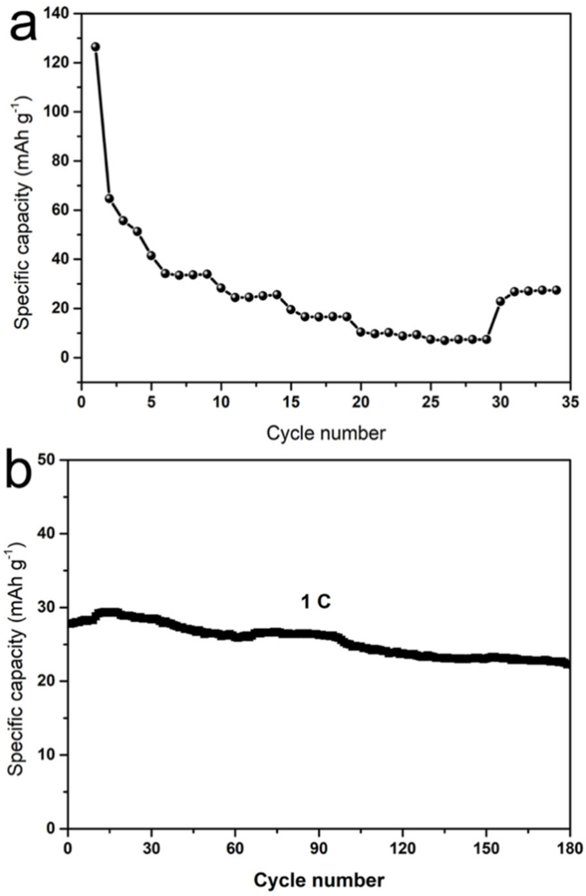

Figure S8. Electrochemical performance of graphene-based anatase electrode under successive incubation at 37 °C and 80 °C. (a) Rate capability of electrodes at various current rates. (b) Cycling performance of electrodes at a current rate of 1C.
